# Exploring the relationship between the reduction of floor microbial burden and the impact on healthcare-associated infections

**DOI:** 10.1017/ash.2023.324

**Published:** 2023-09-29

**Authors:** Caitlin Crews-Stowe, Elizabeth Lambert, Lori Berthelot, Katherine Baumgarten

## Abstract

**Background:** Healthcare floors are a vehicle and/or source for potential pathogens that cause healthcare associated infections, and hospital floors are often heavily contaminated with pathogens such as *Clostridioides difficile* and methicillin-resistant *Staphylococcus aureus*. However, definitive research linking reductions in floor burden to reductions in HAIs has not yet been established. We sought to evaluate emerging technology for continuous disinfection and its potential impact on HAIs. This study was designed to explore the potential relationship between the reduction of microbial burden of floors and healthcare associated infections. **Methods:** A prospective study was conducted in a 22-bed medical-surgical intensive care unit in a 180-bed suburban hospital near New Orleans, Louisiana, from November 2021 to June 2022. Using sterile, premoistened sponges, samples were collected from the floors of 10 areas throughout the unit including 2 nurses’ stations, the physician charting area, and 7 patient rooms. The advanced photocatalytic oxidation (aPCO) equipment was then installed in the HVAC ductwork throughout the ICU and activated. Environmental surface sampling of the same floor surfaces was then repeated every 4 weeks for the first 5 months of the study. HAIs were also tracked throughout the entire study period. The facility’s normal cleaning floor protocols using a neutralizing floor cleaner were unchanged and followed during the study. Changes in surface burden were calculated using a repeated-methods ANOVA with post hoc analyses as appropriate. Rates of healthcare associated infections were compared using χ^2^ analyses. **Results:** Overall, there was a 99.6% statistically significant decrease in floor environmental surface burden from the baseline to the final postactivation test (Fig. 1). The average colony forming unit count (CFU) decreased from 318,850 CFU per 100 cm^2^ to just 2,988 CFU per 100 cm^2^. The unit also saw a statistically significant decrease in publicly reported healthcare associated infections (HO-MRSA, CLABSI, HO-CDI) during the study period compared to the same period a year prior and in the 6 months immediately prior to the beginning of the study (Fig. 2). **Conclusions:** Advanced photocatalytic oxidation technology resulted in a reduction of microbial burden on the floors of a high-traffic intensive care unit. Statistically significant decreases in healthcare-associated infections was also seen. This study highlights a novel aPCO technology and its efficacy at reducing microbial burden and healthcare-associated infections despite no change in practice.

**Figure:**
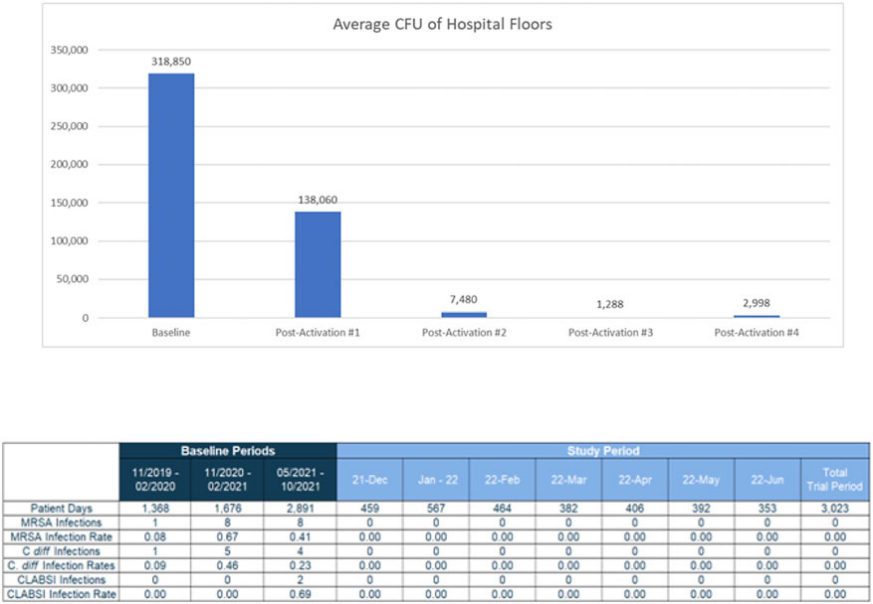

**Disclosures:** None

